# Items of General Interest

**Published:** 1915-02

**Authors:** 


### Items of General Interest.

Physicians of Houston, in liberal number, refused to "concur" in the verdict of the board of censors in condemning "Damaged Goods," but after seeing a private performance a petition was presented to the mayor asking that the films be shown.

The Asheville (N. C.) Ministerial Association passed resolutions January 4, 1915, giving cordial endorsement to a bill before the present session of the Legislature looking to throwing greater restrictions around marriages in North Carolina.

Dr. Wood Hutchings, at the recent meeting of the American Health Association, which was held in Jacksonville, Fla., created quite a sensation by advocating the abolition of practically all the schoolhouses in this part of the country. He said that three-quarters of all necessary things taught in public schools could better be taught out of doors.

Hundreds of thousands of anti-typhoid capsules are now being sent to the French army from the Pasteur Institute in Paris. They are entirely new enterovaccine, discovered by the famous chemist, Adolph Lumiere, and unlike other anti-typhoid vaccines, can be absorbed on the battlefield without feverishness. Every soldier is served with twenty-eight capsules, each containing ten billion of these bacteria, and he swallows four capsules daily for a week. Experiments with 10,000 capsules show immunity from typhoid for a minimum period of three years.

Sir William Osler has astonished the public by his announcement that of 700 wounded who reached one English hospital, only one died. And while it is to be noted that the most serious cases would not be transported across the channel to England, and that other hospitals might not give anything like as good figures, nevertheless, the statement is remarkable. Of course its truth, coming from Sir William Osler, is not to be doubted.

Dr. Morris Booth Miller of Philadelphia, in discussing this matter, made a statement that shows the advances recently made in surgery to a striking degree. In one of the leading hospitals of Philadelphia, during the year just passed, there were performed

1800 surgical operations. Some of these were comparatively minor cases, but a large percentage of the number were most serious major operations. Only twelve of the persons operated on died.

Dr. Geo. M. Converse of the United States Public Health Service returned after three years spent in Iquitos, Peru, whither he went at the request of the Peruvian government to ascertain why the inhabitants were dying at the rate of 50 per 1000 per annum and why virtually the entire population was always either sick or ailing. He found the city very dirty and immediately instituted a cleaning up. There was no hospital, but before he left Dr. Converse had succeeded in reducing the death rate to 21 per 1000 and he had established a well equipped clinic, which, he says, at the end of ten years will have succeeded in saving the lives of about 4000 of the afflicted inhabitants.

Recently there has been organized in New York a society which is known as "The American Society for the Control of Cancer." The purpose of this society is: "To disseminate knowledge concerning the symptoms, diagnosis, treatment and prevention of cancer, to investigate the conditions under which cancer is found and to compile statistics in regard thereto."

The society has received the official approval of the American Medical Association, the American Surgical Association, the American Gynecological Society, the Clinical Congress of Surgeons, the Western and Southern Surgical Associations and the various special medical societies of national scope which together constitute the American Congress of Physicians and Surgeons. Numerous State and local medical societies have also given their endorsement and their active assistance in the work. When we face the fact that 75,000 persons die each year in the United States of this dreadful disease, we cannot help but wonder why something definite has not been done before.

The Public Health Experts have started a nation-wide campaign against malaria. In Florida, Alabama, Georgia, Virginia, North Carolina, Louisiana, Arkansas, Pennsylvania, Tennessee, Georgia, Connecticut, and South Carolina these officials have been working. Educational bulletins are prepared and given wide circulation. Unique among the features of the campaign is a sort of anti-malarial catechism for use by school children.

Mabel McCalmont, hospital consultant, is worth $50 a day to the State of New York, so the department of efficiency and economy has decided.

Professor Laveran has announced a discovery which may prove of great value in the European war. It is a specific for Asiatic cholera, which has been reported to be very prevalent in the Austrian army. Two young scholars in Professor Laveran's institute have demonstrated that salts of lanthanum and thorium destroy the germs of Asiatic cholera. The cure was demonstrated on monkeys which were infected with the cholera and six hours later were dosed with sulphate of lanthanum. They were cured rapidly, while other monkeys which did not receive the dose of salts died in from forty to forty-eight hours.

Amazing unsanitary conditions in rural schools disclosed in investigation by the United States Public Health Service in Eastern Tennessee and Northern Georgia will result in a nation-wide campaign for improved hygiene in the country schoolhouses, it is announced.

Surgeon General Rupert Blue declares that the pollution of the great lakes and tributary rivers is becoming a serious menace. He points out that about 16,000,000 passengers are carried each year over the great lakes and that more than 1600 vessels use these waters. "It becomes apparent, therefore," Dr. Blue declared, "that these inland vessels play an important role in the high typhoid fever rate in the United States."

The law passed by the Thirty-third Legislature giving jurisdiction over lunacy cases to a board of physicians and thus taking it out of the hands of the customary jury, has been declared unconstitutional by Judge William Masterson of the Fifty-fifth district court. In making this ruling, Judge Masterson declared that any law that deprived a citizen of his liberty without due process of law violated the sacred fundamental precepts of American freedom and law. Under the new State law a man charged with lunacy may be arrested and sent to jail and the insane asylum on the authority of a board of physicians to which the law gives supreme jurisdiction. The Constitution of the State and nation says, in effect, that every man is entitled to the right of a trial before a jury before his liberty can be taken away from him.

